# Co‐occurrence of glial fibrillary acidic protein astrocytopathy in a patient with Leber's hereditary optic neuropathy due to *DNAJC30* mutations

**DOI:** 10.1111/ene.16344

**Published:** 2024-05-17

**Authors:** Maria Pia Giannoccaro, Luana Morelli, Fortuna Ricciardiello, Vincenzo Donadio, Fiorina Bartiromo, Caterina Tonon, Michele Carbonelli, Giulia Amore, Valerio Carelli, Rocco Liguori, Chiara La Morgia

**Affiliations:** ^1^ IRCCS Istituto delle Scienze Neurologiche di Bologna Bologna Italy; ^2^ Dipartimento di Scienze Biomediche e Neuromotorie Università di Bologna Bologna Italy; ^3^ Ophthalmology Unit, Dipartimento di Scienze Mediche e Chirurgiche Alma Mater Studiorum University of Bologna Bologna Italy; ^4^ IRCCS Azienda Ospedaliero‐Universitaria di Bologna Bologna Italy

**Keywords:** area postrema syndrome, GFAP antibodies, GFAP astrocytopathy, Leber's hereditary optic neuropathy, neuromyelitis optica, NMOSD

## Abstract

Leber's hereditary optic neuropathy (LHON) is a mitochondrial disease characterized by visual loss, and rarely associated with extraocular manifestations including multiple sclerosis‐like lesions. The association of LHON and neuromyelitis optica spectrum disorders has rarely been reported. Here is reported a case of glial fibrillary acidic protein astrocytopathy presenting with area postrema syndrome in a patient with previously diagnosed recessive LHON due to mutations in the nuclear gene *DNAJC30*. This case emphasizes the necessity of extensive investigations for other treatable conditions in patients with LHON and otherwise unexplained extraocular involvement and the possibility that also visual symptoms can respond to immune therapy.

## INTRODUCTION

Area postrema syndrome (APS) is one of the clinical presentations of neuromyelitis optica spectrum disorders (NMOSD) and has rarely been associated with glial fibrillary acidic protein (GFAP) antibodies [1]. Leber's hereditary optic neuropathy (LHON) and NMOSD might share some clinical overlap, such as severe visual impairment and myelopathy, and LHON should be considered in the diagnostic workup of patients with recurrent optic neuritis as well as some cases of longitudinally extensive spinal cord lesions [2].

Here a case of GFAP astrocytopathy presenting with APS is reported in a patient with LHON.

## CASE PRESENTATION

A 21‐year‐old Caucasian man presented to the emergency department with a 1‐month history of nausea, vomiting, vertigo and gait instability. Family history was relevant for autoimmune disorders including type 1 diabetes and celiac disease (sister), systemic lupus erythematosus (maternal grandmother), Hashimoto's thyroiditis (two maternal cousins) and dermatomyositis (maternal cousin).

**FIGURE 1:**
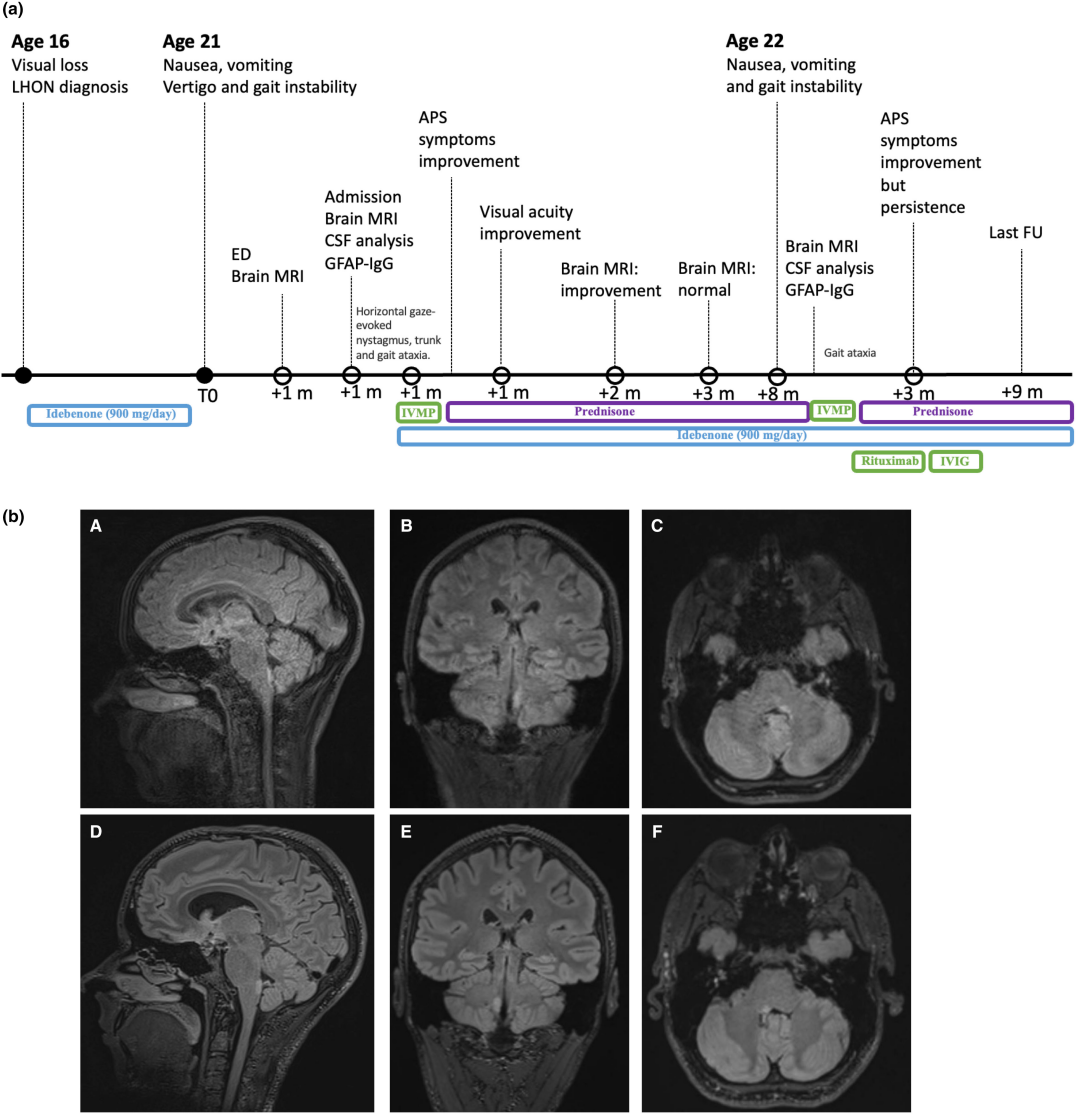
(a) Case timeline. (b) Brain MRI features during the first (A–C) and the second (D–F) episode, showing the presence of a right (A–C) and left (D–F) APS hyperintense lesion in T2 weighted images. APS, area postrema syndrome; CSF, cerebrospinal fluid; ED, emergency department; IVIG, intravenous immunoglobulin; IVMP, intravenous methylprednisolone.

At age 16, he developed subacute visual loss of the left eye (LE) and, 1 week later, of the right eye (RE). He underwent extensive investigations including myelin oligodendrocyte glycoprotein (MOG) immunogobulin G (IgG), aquaporin‐4 (AQP4) IgG and brain magnetic resonance imaging (MRI), all normal. Visual acuity (VA) progressively worsened, reaching a nadir 9 months after onset (RE 2/100; LE counting finger (CF)). Complete mitochondrial DNA (mtDNA) sequence ruled out the presence of mtDNA variants, whereas exome sequencing showed the presence of homozygous disease‐causing variants (c.152A>G, p.Tyr51Cys) in the nuclear gene *DNAJC30* [3] and he was diagnosed as recessive LHON. Idebenone therapy was started (900 mg/day), continued for 4 years until a plateau was achieved and stopped about 1 month before the current episode. A modest VA improvement was observed after 2 years of therapy (RE 8/100; LE 8/100) (Figure 1a).

During the current episode, he underwent a brain MRI, which disclosed a hyperintense lesion of the fourth ventricle's floor on T2 fluid‐attenuated inversion recovery images. Neurological examination demonstrated bilateral horizontal gaze‐evoked nystagmus, trunk ataxia, brisk and symmetrical tendon reflexes, and mild gait ataxia. Laboratory investigations were normal. Cerebrospinal fluid (CSF) analysis showed increased IgG index (0.68, normal value <0.66) and oligoclonal bands. A new brain MRI confirmed a hyperintense T2 lesion of the area postrema (AP) with mild contrast enhancement (Figure 1b, A–C). A spinal MRI was normal. Serum antinuclear antibody spectrum screening was unremarkable. Serum and CSF screened by live cell‐based assay were negative for AQP4‐IgG and MOG‐IgG. However, a fixed cell‐based assay and a tissue‐based assay were positive for GFAP‐IgG in both serum (1:3200) and CSF (1:20). Other neuronal antibodies were negative. Pulse methylprednisolone treatment with subsequent oral tapering led to progressive symptom regression and disappearance of the AP lesion. Moreover, unexpectedly, the patient had VA improvement (RE 2.5/10; LE 2/10) after 2 months of steroid therapy up to 6.3/10 bilaterally about 1 year later.

Fourteen months after onset, whilst still on oral steroid (12.5 mg/day), he had a new episode of nausea, vomiting and left lateropulsion. Brain MRI showed a new AP lesion (Figure 1b, D–F) displaying contrast enhancement. CSF analysis showed increased IgGs and oligoclonal bands. GFAP‐IgG was still positive in serum (1:1600) and CSF (1:10). He underwent a new course of methylprednisolone therapy, followed by rituximab, oral steroid tapering and intravenous immunoglobulin therapy until recovery. At the last follow‐up, 12 months later, no relapse had occurred.

## DISCUSSION

This is the first case of concomitant GFAP astrocytopathy and recessive *DNAJC30* LHON. To date, a few cases of concomitant seropositive NMOSD and LHON have been reported, all associated with mtDNA mutations and complex I dysfunction. This is the first case of a recessive LHON case associated with autoimmunity. *DNAJC30* mutations have been reported to affect complex I turn‐over and function and are associated also with Leigh syndrome [3]. The pathogenesis of GFAP astrocytopathy is unclear. Antibodies against the intracellular GFAP protein are unlikely to be pathogenic and are probably a biomarker of a cytotoxic T‐cell‐mediated autoimmune response. Given the rarity of both LHON and NMOSD it is tantalizing to speculate on a possible pathogenic link between these disorders. In fact, growing evidence suggests a possible role of mtDNA in triggering secondary autoimmunity, through the activation of the innate immune system, as well as systemic inflammation [4, 5].

Remarkably, in our case, an improvement of the VA was observed upon prolonged steroid therapy, years after LHON onset, suggesting a possible role of inflammation in the visual impairment due to mitochondrial dysfunction. Indeed, variable degrees of responses to immunotherapies have been reported in some mitochondrial patients [5].

Despite the intriguing possibility of a relation between LHON‐related mutations and autoimmunity, further experimental data are needed to confirm this hypothesis. In conclusion, this case highlights the possibility of concomitant NMOSD and of potential response to immunotherapy also of visual symptoms in LHON.

## AUTHOR CONTRIBUTIONS

**Maria Pia Giannoccaro:** Conceptualization; methodology; formal analysis; data curation; visualization; writing – original draft; investigation. **Luana Morelli:** Investigation; data curation; writing – review and editing. **Fortuna Ricciardiello:** Investigation; formal analysis; writing – review and editing. **Vincenzo Donadio:** Data curation; writing – review and editing. **Fiorina Bartiromo:** Investigation; writing – review and editing. **Caterina Tonon:** Investigation; writing – review and editing. **Michele Carbonelli:** Investigation; writing – review and editing. **Giulia Amore:** Investigation; writing – review and editing. **Valerio Carelli:** Writing – review and editing; supervision. **Rocco Liguori:** Supervision; writing – review and editing. **Chiara La Morgia:** Data curation; supervision; conceptualization; writing – review and editing.

## FUNDING INFORMATION

The author(s) received no financial support for the research, authorship and/or publication of this article.

## CONFLICT OF INTEREST STATEMENT

The author(s) declared no potential conflicts of interest with respect to the research, authorship and/or publication of this article.

## ETHICS STATEMENT

Our institution does not require ethical approval for reporting individual cases. Written informed consent was obtained from the patient for his anonymized information to be published in this article.

## Data Availability

Data sharing is not applicable to this article as no datasets were generated or analysed during the current study.
